# In situ analysis of hepatitis B virus (HBV) antigen and DNA in HBV-induced hepatocellular carcinoma

**DOI:** 10.1186/s13000-022-01194-8

**Published:** 2022-01-16

**Authors:** Ye Zheng, Mingzhu Xu, Dong Zeng, Haitao Tong, Yuhan Shi, Yanling Feng, Xiaonan Zhang

**Affiliations:** 1 Shanghai Public Health Clinical Center, Fudan University, Shanghai, China; 2 Centre for Research in Therapeutic Solutions, Faculty of Science and Technology, University of Canberra, Canberra, ACT Australia

**Keywords:** HBV-related HCC, HBV DNA, HBsAg, In situ hybridization, Integration

## Abstract

**Aims:**

Hepatitis B Virus (HBV) infection is the major risk factor for hepatocellular carcinoma (HCC) in East Asia. Here we aimed to further investigate the abundance of viral antigen and DNA within HBV-related HCC and surrounding tissues at histological level.

**Method:**

In addition to routine histopathology, in situ hybridization (ISH) of HBV DNA and immunohistochemistry (IHC) of HBsAg were performed in tissues from 131 HBsAg-positive HCC patients undergoing liver resection. Serum α-fetoprotein together with basic biochemical and immunological parameter was also measured.

**Results:**

Overall, the ISH of HBV DNA and IHC of HBsAg showed 31.3% and 92.9% positive rate respectively (*p* < 0.0001). The level of correlation between these two markers was much more significant in tumor (*p* < 0.0001) than in tumor-surrounding tissue (*p* = 0.01). HBsAg exhibited a much higher positive rate in tumor-adjacent tissue than in tumor tissue (86.6% versus 29.9%, *p* < 0.0001) with significantly different staining pattern. By contrast, the positive rate of HBV DNA ISH was comparable in tumor and surrounding tissue (17.6% versus 22.9%, *p* = 0.36). Yet the HBV DNA signal in tumor tissue showed predominant nuclear localization (87.0%) whereas staining pattern in adjacent tissue was mixed (43.3% nuclear localization, *p* = 0.0015). Finally, no significant association between intra-tumor HBV DNA/HBsAg positivity and major histological markers (microvascular invasion, tumor differentiation, etc.) or recurrence after surgery was observed.

**Conclusions:**

These data confirmed the largely integrated state of HBV DNA, weaker expression and altered localization of surface antigen in tumor compared with surrounding tissue. The strikingly different prevalence and localization of HBsAg and HBV DNA reflected the complex and heterogeneous mechanisms leading to HBV-induced tumorigenesis.

**Supplementary Information:**

The online version contains supplementary material available at 10.1186/s13000-022-01194-8.

## Introduction

Hepatocellular carcinoma (HCC) is one of the most common cause of cancer and second most frequent cause of cancer-related death globally with 854,000 new cases and 810,000 deaths per year [1]. The occurrence of HCC is the highest in East Asia (> 20/100,000) and sub-Sahara Africa, 60% of which is due to Hepatitis B Virus (HBV) [1, 2]. HBV-induced carcinogenesis involves a series of events such as viral integration and subsequent genomic instability, oncogenic effects of viral proteins (HBx, preS/S envelope proteins, etc.) and sustained cycles of necroinflammation-regeneration [3, 4]. Although dispensable for the viral life-cycle, HBV DNA integration is widely accepted as the direct oncogenic event contributing to HCC. The most frequently reported integration loci include TERT, CCNE1, and MLL4 etc. [5, 6]. It constitutes the initial strike promoting clonal expansion and also triggers larger-scale chromosomal rearrangement as a result of genome instability. In addition, the introduced HBV DNA also express wildtype and mutated/truncated viral proteins (HBx and HBsAg) which further drives dysplastic nodules into progressed carcinoma [7].

Although previous studies had been conducted to analyze the viral DNA in normal and tumor tissue in all stages of HBV-related diseases [5, 6, 8–14], there is generally a lack of molecular investigations taking histological features into account. We recently developed a sensitive in situ hybridization (ISH) assay for HBV DNA and revealed a mosaic distribution of viral DNA and antigens at single-cell level [15]. It is also highly correlative to the vigor of viral replication in chronic hepatitis B [16]. Here, by using this methodology, we aimed to further analyze the histological features of HBV DNA together with viral surface protein in HCC patients.

## Materials and methods

### Patients

This study enrolled 131 HBV-related (serum HBsAg positive) HCC patients admitted into the Shanghai Public Health Clinical Center (2016–2019) who underwent partial surgical resection of the liver. They were followed up for one year after surgery for recurrence, complications or death. The pathological diagnosis for the patients was in accordance with the WHO classification of Digestive System tumors (5th edition, 2019).

### Pathological assessment

The liver tumors along with surrounding tissues were routinely formalin-fixed and paraffin-embedded. The tissue paraffin sections (8 μm thick) were subjected to hematoxylin and eosin (H&E) staining, reticulin staining and immunohistochemistry of HBsAg together with other key markers (Hep-par1, GPC-3, GS, Ki-67, Hsp70). Edmondson-Steiner grade (differentiation) and microvascular invasion (MVI) grade were assessed. The necro-inflammation and fibrosis were assessed by Scheuer score.

Immunohistochemistry was performed using Leica BOND automatic stainer. The staining results were examined in comparison with adjacent HE sections in order to evaluate the signal intensity in tumor and surrounding areas. The IHC results were scored as 0, 1, 2, 3 corresponding to proportion of the immunolabelled cells of 0, < 5, 5–20% and > 20%, respectively.

### Biochemical, serologic, and virological parameters

Serum samples were obtained before surgery. Serum HBsAg, HBeAg, anti-HBe were measured by chemiluminescence microparticle immunoassay (CMIA) (Abbott, USA). Serum HBV DNA was measured using quantitative PCR assay (Sansure, China) with a detection range of 5 × 10^2^ to 2 × 10^9^ IU/mL. Serum alanine transferase (ALT) and other biochemical parameters were measured by Abbott Accelerator a3600 full-automatic biochemical analyzer (Abbott, USA).

### In situ hybridization of HBV DNA

The procedures of pretreatment, probeset hybridization and amplification were based on our previous study [16] and performed using the ViewRNA ISH Tissue Assay (Thermo Fisher, Fremont, CA). The tissue sections were routinely dewaxed and rehydrated, followed with antigen retrieval, protease digestion and refixation with 4%formaldehyde in PBS for five minutes. The HBV DNA probe (VF6–20095) was designed to target the minus strand sequence (nt2959–837) conserved from genotype A to D. After probe hybridization and signal amplification, sections were stained with NBT/BCIP (Roche) in developing solution at 37 °C for 2 h. Sections were then counterstained with Sirius red and washed with water before air-dry and mounting. Rigorous controls were included (positive control slides, no probe ISH control experiments on adjacent slides) were included to ensure the specificity of the ISH assays. The ISH results were examined in comparison with HE staining results in adjacent sections and a score of 0-to-3 was given to tumor and surrounding areas with a standard similar to IHC.

### Ethics statement

This study was conducted in accordance with the guidelines of the 2013 Declaration of Helsinki and approved by the independent ethics committee of Shanghai Public Health Clinical Center, Fudan University (2021-S003–01).

### Statistical analysis

Statistical analyses were performed using Medcalc version 15.8 (Mariakerke, Belgium) and the Prism 6 (Graphpad, USA). For continuous variables, median and interquartile range (IQR) was reported. Percentage was used to report categorical parameters. Comparisons of ISH/IHC grades between paired tumor and surrounding were done via Rank-sum test (Wilcoxon test), Chi-square test and Fisher’s exact test for categorical data. Significance was assumed at *p* < 0.05 for all tests.

## Results

The basic demographic features of the enrollment were shown in Table 1. The age range of the patients was 48–62 years (median 55 yrs). The male-to-female ratio was 4.59 (male 107, female 24), similar to previous epidemiological reports [2]. These patients had a medium α-fetoprotein level of 39.9 ng/ml (interquartile range, 8.0–522.2 ng/ml) and a medium ALT level of 31.0 U/L (interquartile range, 21.0–49.3 U/L). The positive rates of immunohistochemical results of liver tumor diagnostic markers (Hep-par1, GPC-3, GS, Ki-67, Hsp70) are all higher than 95% (Table 1). The majority of the cases had advanced fibrosis or cirrhosis with 74.8% of them having Scheuer stage of grade 4 and 73.3% having necro-inflammation score > =2.

**Table 1:** Demographic and histologic characteristics of HCC patients

Characteristics	*n* = 131
**Age (yrs)**	55 (48–62)
**Gender**	
Female (%)	24 (18.32%)
Male (%)	107 (81.68%)
**HBeAg**	
Positive(%)	26 (19.74%)
Negative(%)	77 (58.77%)
Not available(%)	28 (21.37%)
**Serum HBsAg**^**a**^ **(IU/mL)**	
<250	35 (33.98%)
>250	68 (66.02%)
**Serum HBV DNA**^**b**^ **(IU/mL)**	
<500	78 (68.42%)
>500	36 (31.58%)
**Diagnostic markers**	
Ki-67	131 (100%)
Hep-par1	127 (96.95%)
GPC-3	128 (97.71%)
GS^c^	125 (96.15%)
HSP70^d^	124 (96.88%)
**α-fetoprotein (ng/ml)**	39.9 (8.0–522.2)
**Alanine transferase ALT (U/L)**	31.0 (21.0–49.3)
**Necro-Inflammation (G1,G2,G3,NA)**	17:66:30:18
**Fibrosis (S1,S2,S3,S4,NA)**	5:6:4:98:18
**Tumor Differentiation (1,2,3)**	12:98:21
**MVI** (M0,M1,M2)	60: 44: 27
**Satellite nodule**	7 (5.34%)
**Nodule in nodule**	27 (20.61%)
**Recurrence**	
Yes	68 (51.90%)
No	61 (46.56%)
death	2 (1.52%)

**Fig. 1:**
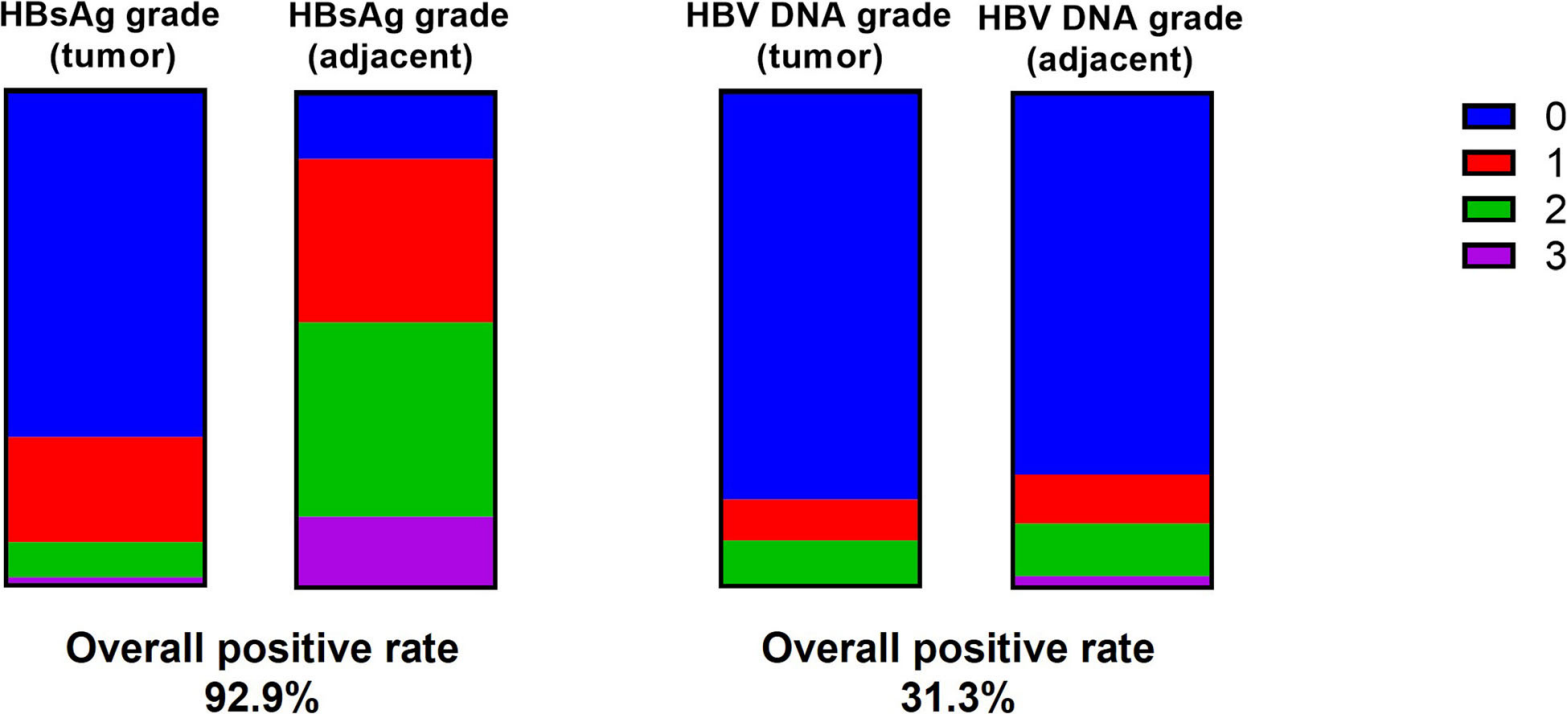
Frequency of HBsAg IHC and HBV DNA ISH grades in liver tumor and adjacent tissues

**Fig. 2:**
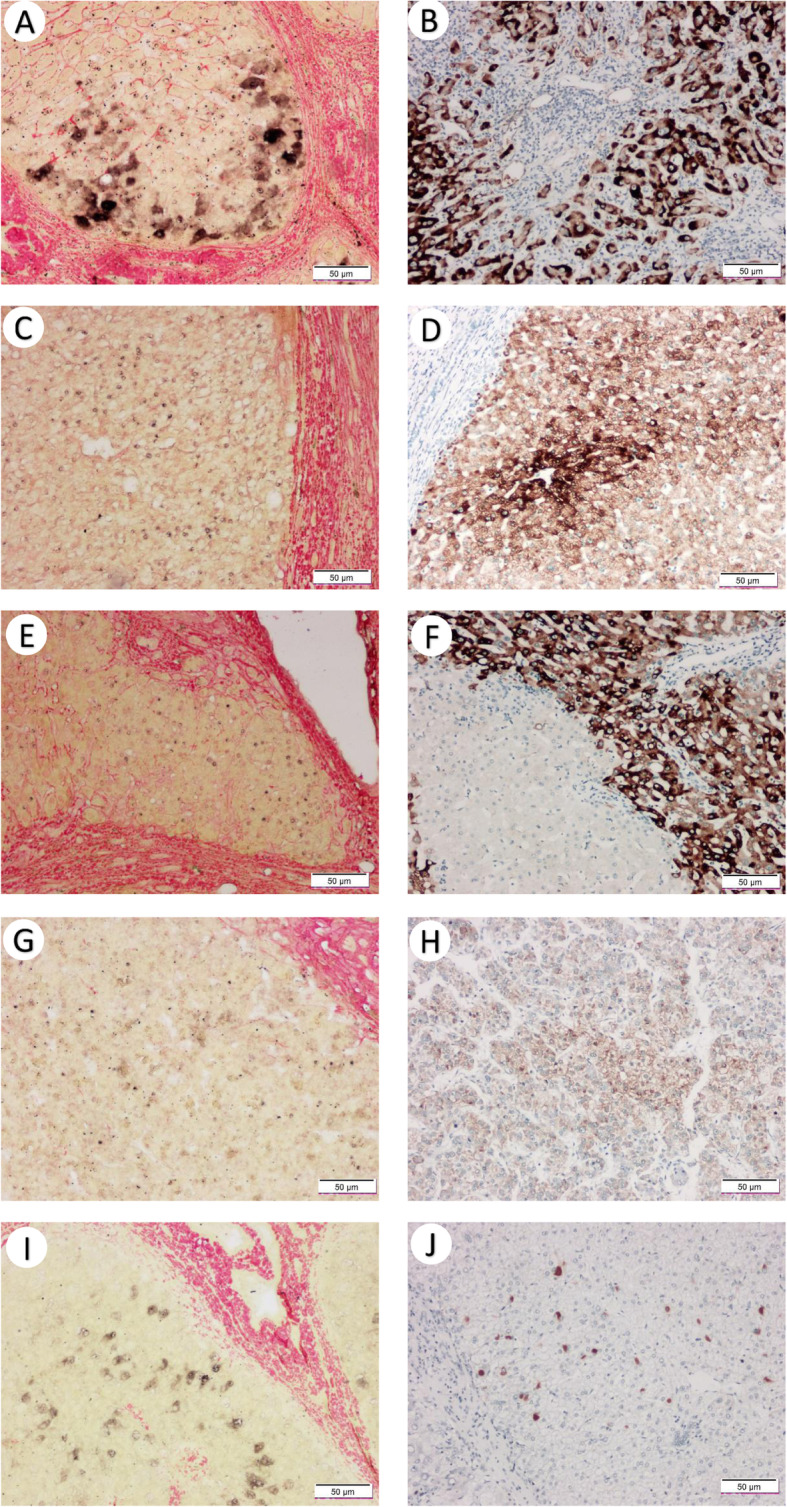
Typical results of HBV DNA ISH (**A,C,E,G,I**) and HBsAg IHC (**B,D,F,H,J**). (A-B) Intense HBsAg and HBV DNA signal observed in only the tumor-surrounding tissues of a patient. In another two patients (C-F and G-J), HBV DNA and HBsAg can be observed in both surrounding tissue (E-F, I-J) and within tumor (C-D, G-H)

Data expressed as the median (interquartile range)

a. 28 cases missing serum HBsAg information;

b. 17 cases missing serum HBV DNA information;

c. 1 cases missing IHC GS information;

d. 3 case missing IHC Hsp70 information;

Among these 131 cases, the overall positive rate of HBsAg IHC (127 cases, four cases missing) was 92.9%, significantly higher than that of HBV DNA ISH 31.3% (*p* < 0.0001, Fisher’s exact test, Fig. 1). Closer inspection of positive staining results revealed that HBsAg showed significantly higher positivity in surrounding tissues than in tumor (86.6% versus 29.9%, *p* < 0.0001, Fisher’s exact test, Table 2). The same trend was found in the paired-analysis of IHC grades (p < 0.0001, Wilcoxon test, Table 2, Fig. 1). The pattern of HBsAg in tumor surrounding tissue was usually intensely stained within a cluster of hepatocytes (Fig. 2B, F). In tumor tissues, HBsAg usually exhibited a weaker and membranous expression (Fig. 2D, H). On the other hand, we found that the grades of HBV DNA ISH in tumor and surrounding tissue were not statistically different (*p* = 0.09, Wilcoxon test, Table 2). However, the majority of HBV DNA signal in tumor was within the nuclei (87.0%) whereas a much lower rate of nuclear localization was found in adjacent tissue (43.3%, *p* = 0.0015, Fisher’s exact test, Table 3, Fig. 2C, G). Indeed, the typical cytoplasmic distribution pattern of HBV DNA in tumor-adjacent tissues (Fig. 2A, I) was indistinguishable from that of chronic hepatitis B [15]. In many cases, these signals were often in proximity to collagen fibers as shown by Sirius red staining (Fig. 2).

**Table 2:** The summary of molecular pathological results in tumor and surrounding tissues

	0	1	2	3	Positive rate (%)	*p* value^b^
HBsAg IHC grade in tumor tissue^a^	89	27	9	2	29.9	
HBsAg IHC grade in adjacent tissue^a^	17	42	50	18	86.6	< 0.0001
HBV DNA ISH grade in tumor tissue	108	11	12	0	17.6	
HBV DNA ISH grade in adjacent tissue	101	13	14	3	22.9	0.09

a. Four cases missing IHC information

b. Rank-sum test (Wilcoxon test)

**Table 3:** The pattern of HBV DNA subcellular localization in tumor and surrounding tissues

	Nuclear	Cytoplasmic	*p* value^a^
HBV DNA localization in tumor tissue	20	3	
HBV DNA localization in adjacent tissue	13	17	0.0015

a, Fisher’s exact test

We continued to assess the relationship between circulating and in situ virological markers. We found that serum HBV DNA was not significantly higher when comparing total ISH positive cases with negative cases (*p* = 0.3939, Mann-Whitney U test, Supplementary Fig. 1A, left). However, significant difference in serum viral load was found between DNA positive and negative cases in adjacent tissues (*p* = 0.0335, Mann-Whitney U test, Supplementary Fig. 1A, middle). This is in accordance with our previous observations in chronically infected patients [16, 17]. By contrast, the intra-tumor ISH positive cases did not show higher viral load compared with negative ones (*p* = 0.2358, Mann-Whitney U test, Supplementary Fig. 1A, right). For serum HBsAg, due to the complexity of its source in HCC, one from cccDNA transcription (that is, viral replication), and the other from HBV integration, we were unable to find significant relationship between HBsAg titre and in situ prevalence of HBV DNA (Supplementary Fig. 1B, left) or in situ expression of HBsAg (Supplementary Fig. 1B, right).

We next evaluated the relationship between in situ markers. The HBV DNA ISH and HBsAg IHC were found to be weakly correlated, as none of the HBsAg negative patients (9 cases) were found to be HBV DNA positive whereas 41 of the 118 cases of HBsAg positive cases were DNA positive (*p* = 0.03, Fisher’s exact test, Supplementary Table 1). However, when analyzing the positivity of these assays in tumor and non-tumor regions separately, a stronger association was found within tumor (*p* < 0.0001, Fisher’s exact test, Supplementary Table 2) compared with that in tumor-surrounding area (*p* = 0.0118, Fisher’s exact test, Supplementary Table 3).

There were no significant associations between the positivity of HBsAg or HBV DNA and the grade of tumor differentiation (HBsAg, *p* = 0.16, HBV DNA, *p* = 0.32, Mann-Whitney test).

We then probed the possible relationship between intra-tumor HBV HBsAg or HBV DNA and major histological (microvascular invasion, satellite nodule, nodule-in-nodule) and prognostic (recurrence after resection) parameters. Due to the very high positive rate of Ki-67, Hep-par1, GPC-3, Ki-67, and GS (> 95%), we could not find any correlation with viral DNA or surface antigen within tumor. Nor did we find significant links with microvascular invasion, satellite nodule or nodule in nodule (Supplementary Table 4, 5). Finally, recurrence rates were not significantly different among patients with positive or negative intra-tumor HBV DNA/antigen.

## Discussion

As an aberrant by-product of hepadnaviral replication, double-stranded linear DNA can form during chronic hepatitis B infection which serves as the template for random integration into host genome by nonhomologous recombination [13]. The excess of such DNA during chronic hepatitis greatly increases the rate of DNA insertion in somatic cell, facilitating the outgrowth of cell clones with dysregulated pro-proliferative/cancer suppressor genes [5, 6, 8]. In addition, the integrated DNA drives the expression of viral proteins such as HBx [18], which further potentiates the proliferation of the dysplastic clones and leads to progressed hepatocellular carcinoma.

A number of studies had been undertaken to evaluate the abundance of viral antigen and DNA in HBV-positive HCC samples. Two studies analyzed the form of viral DNA in HCC samples and both found higher incidence of viral DNA integration in tumor than in non-tumor tissue whereas free replicative forms were more frequently found in non-tumor [10, 19]. Indeed, we have found that although the positive rate of viral DNA was comparable in tumor and non-tumor tissue, there exists a significant difference in its localization. The predominant nuclear pattern in tumor strongly supports the integrated state of these DNA. The significantly lower positive rate of viral DNA (31.3%) in our study compared with Southern blot assay (> 80%) [10, 19] is probably caused by the relatively lower sensitivity of ISH assay. In addition, studies have shown that the length of the integrated sequence ranges from 28 bp to the full length [20], and our probe was designed to target the HBsAg coding region (nt2959–837), there may be undetected partial integration. In terms of HBsAg expression, we found a lower prevalence of HBsAg in tumor tissues and lower IHC grades in paired-analysis. This suggests that although viral integration is wide-spread in HBV-related HCC, the expression of viral antigens is mostly down-regulated or silenced. Indeed, Bowyer et al. examined the methylation status of HBV DNA and found that integrated DNA were mostly hypermethylated [21]. A recent report suggested the existence of viral mRNA in HCC samples even though viral antigens were not detectable by conventional methods [22]. This further supported the high prevalence of viral DNA integration but low expression of viral antigen.

In terms of the relationship between HBV DNA ISH and HBsAg IHC results, the limited correlation in tumor-adjacent tissues was expected as our previous work also found low relatedness in chronic hepatitis B [16] due to the ultra-low copy number of viral DNA in HBsAg-rich cells [15]. However, a strong relationship was found in tumor region. We suggest that this is caused by the integrated nature of viral DNA, which is detectable by ISH only when a relatively high copy number is achieved. Indeed, compared with previous Southern blot results [10, 19], many of the ISH negative tumor samples may actually harbor HBV integrants, however HBsAg may not be easily detectable due to the profoundly silenced nature of these sequences [21].

The fact that intra-tumor HBV DNA or HBsAg expression was not associated with the differentiation, microvascular invasion, satellite nodule, or nodule-in-nodule should not be surprising since the development of hepatocellular malignancy is highly variable and heterogeneous. It is the site of the integration, rather the existence of integration itself that has significant impact on the growth of hepatocyte clones. Hence, it is also reasonable to find their lack of association with the recurrence rate.

In conclusion, using in situ hybridization, we re-evaluated the distribution of viral DNA in tumor and non-tumor tissue of HBV-related HCC. Our results confirmed the prevalent HBV DNA integration but much lower expression of viral surface antigen within tumor compared with non-tumor tissue. The varied integration in different cell clones exhibited varied epigenetic feature, growth property and invasiveness of HCC among patients. Although viral antigens were expressed at low level, targeting these epitopes using engineered cytotoxic lymphocytes might be a feasible strategy for recurrent tumor cells after liver transplantation [22].

## Supplementary Information

**Additional file 1: Supplementary Fig. 1.** Correlations between circulating and in situ virological markers. (A) serum viral loads and (B) serum HBsAg titre were compared between HBV DNA ISH positive and negative cases categorized based on signal in total (left), tumor (middle), or adjacent tissue (right).

**Additional file 2: Supplementary. Tables 1–5.**

## Data Availability

All data generated or analyzed during this study are included in this manuscript.

## Abbreviations

HBV: Hepatitis B virus
HCC: hepatocellular carcinoma
ISH: in situ hybridization
IHC: immunohistochemistry
ALT: alaine transferase
