# Supplementary figures and images for: In situ analysis of hepatitis B virus (HBV) antigen and DNA in HBV-induced hepatocellular carcinoma
Source: Diagn Pathol. 2022 Jan 16;17:11. doi: 10.1186/s13000-022-01194-8 (PMC8761330; doi:10.1186/s13000-022-01194-8)

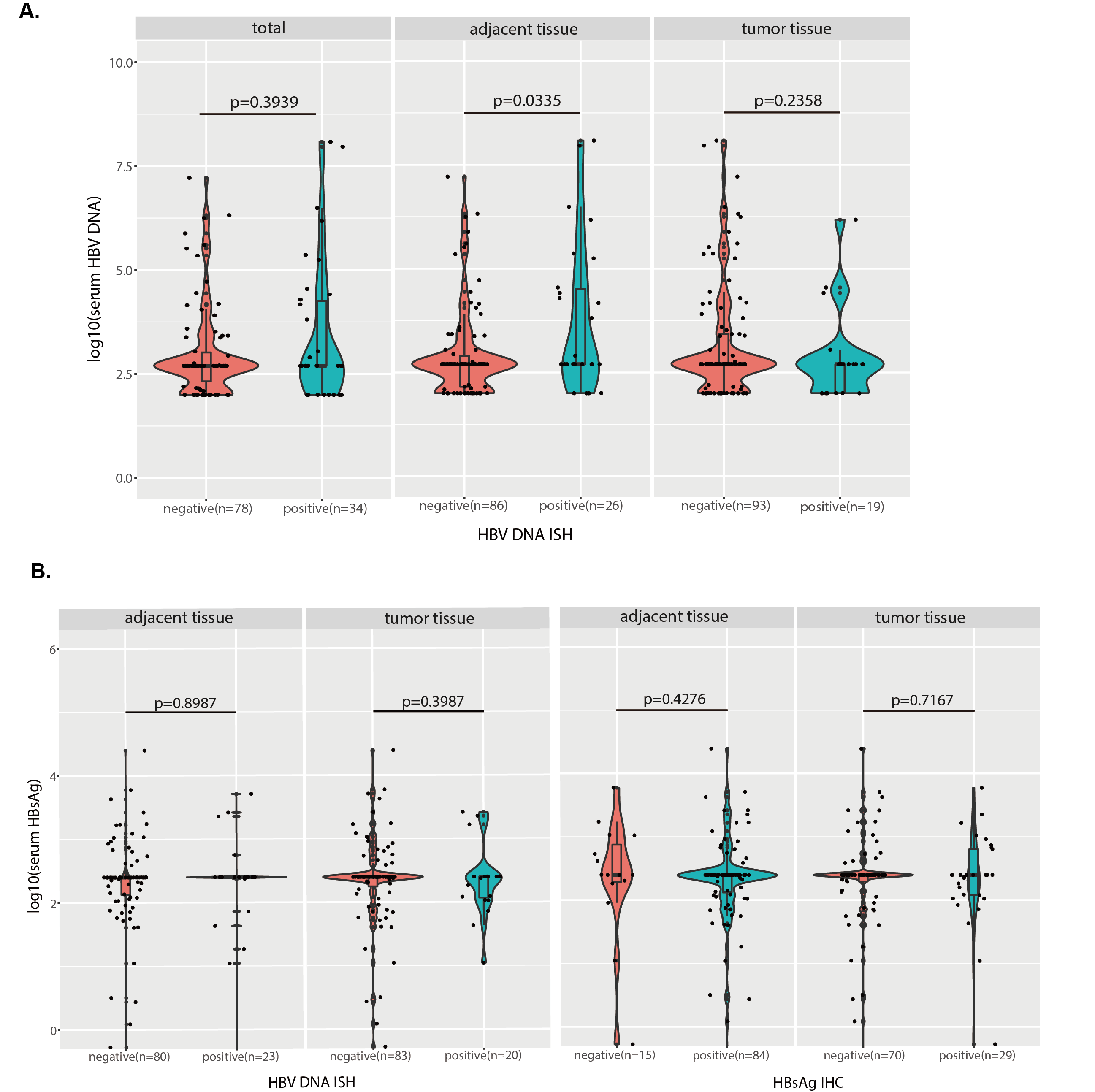

Supplement: Supplementary file 1 — Additional file 1: Supplementary Fig. 1. Correlations between circulating and in situ virological markers. (A) serum viral loads and (B) serum HBsAg titre were compared between HBV DNA ISH positive and negative cases categorized based on signal in total (left), tumor (middle), or adjacent tissue (right). [file 13000_2022_1194_MOESM1_ESM.png]
