# Supplementary material for: In situ analysis of hepatitis B virus (HBV) antigen and DNA in HBV-induced hepatocellular carcinoma
Source: Diagn Pathol. 2022 Jan 16;17:11. doi: 10.1186/s13000-022-01194-8 (PMC8761330; doi:10.1186/s13000-022-01194-8)
Supplement: Supplementary file 2 — Additional file 2: Supplementary. Tables 1–5. [file 13000_2022_1194_MOESM2_ESM.docx]

**Supplementary Table 1. Cross tabulation table of HBsAg IHC and HBV DNA ISH results in the whole section**

|  | HBsAg IHC positive | HBsAg IHC negative | total |
| --- | --- | --- | --- |
| HBV DNA ISH positive | 41 | 0 | 41 |
| HBV DNA ISH negative | 77 | 9 | 86 |
| Total | 118 | 9 | 127^*^ |

p=0.0311 (Fisher’s exact test)

* HBsAg IHC results were not available in four cases.

**Supplementary Table 2. Cross tabulation table of HBsAg IHC and HBV DNA ISH results in tumor**

|  | HBsAg IHC positive | HBsAg IHC negative | total |
| --- | --- | --- | --- |
| HBV DNA ISH positive | 16 | 7 | 23 |
| HBV DNA ISH negative | 22 | 82 | 104 |
| Total | 38 | 89 | 127^*^ |

P<0.0001 (Fisher’s exact test)

* HBsAg IHC results were not available in four cases.

**Supplementary Table 3. Cross tabulation table of HBsAg IHC and HBV DNA ISH results in tumor-surrounding tissue**

|  | HBsAg IHC positive | HBsAg IHC negative | total |
| --- | --- | --- | --- |
| HBV DNA ISH positive | 30 | 0 | 30 |
| HBV DNA ISH negative | 80 | 17 | 97 |
| Total | 110 | 17 | 127^*^ |

P=0.0118 (Fisher’s exact test)

* HBsAg IHC results were not available in four cases.

**Supplementary Table 4. Intra-tumor HBV DNA ISH in correlation with major histologic and prognostic parameters**

|  | ISH positive | ISH negative | Total | p value |
| --- | --- | --- | --- | --- |
| **Hsp70**  positive  negative | 23  0 | 101  4 | 124  4 | >0.9999^a^ |
| **Hep-par1**  positive  negative  **GPC-3**  positive  negative  **Ki-67**  positive  negative  **GS**  positive  negative  **MVI**  M0  M1  M2  **Satellite nodule**  yes  no  **Nodule in nodule**  yes  no  **Recurrence**  yes  no | 23  0  22  1  23  0  22  1  13  7  3  0  23  5  18  11  12 | 104  4  106  2  108  0  103  4  47  37  24  7  101  22  86  57  49 | 127  4  128  3  131  0  125  5  60  44  27  7  124  27  104  68  61 | >0.9999 ^a^  0.4424 ^a^  >0.9999 ^a^  >0.9999 ^a^  0.4589 ^b^  0.3529 ^a^  >0.9999 ^a^  0.6501 ^a^ |

a, Fisher’s exact test

b, Chi-square test

**Supplementary Table 5. Intra-tumor HBsAg IHC in correlation with major histologic and prognostic parameters**

|  | IHC positive | | IHC negative | Total | p value |
| --- | --- | --- | --- | --- | --- |
| **Hsp70**  positive  negative | 37  1 | | 84  3 | 121  4 | >0.9999 ^a^ |
| **Hep-par1**  positive  negative  **GPC-3**  positive  negative  **Ki-67**  positive  negative  **GS**  positive  negative  **MVI**  M0  M1  M2  **Satellite nodule**  yes  no  **Nodule in nodule**  yes  no  **Recurrence**  yes  no | | 38  0  37  1  38  0  36  1  18  10  10  2  36  7  31  16  21 | 85  4  87  2  89  0  85  4  40  33  16  5  84  20  69  49  39 | 123  4  124  3  127  0  121  5  58  43  26  7  120  27  100  65  60 | 0.3160 ^a^  >0.9999 ^a^  >0.9999 ^a^  >0.9999 ^a^  0.3966 ^b^  >0.9999 ^a^  0.8131 ^a^  0.2415 ^a^ |

a, Fisher’s exact test

b, Chi-square test
